# Phytochemical Composition and Antioxidant Capacity of 30 Chinese Teas

**DOI:** 10.3390/antiox8060180

**Published:** 2019-06-18

**Authors:** Guo-Yi Tang, Cai-Ning Zhao, Xiao-Yu Xu, Ren-You Gan, Shi-Yu Cao, Qing Liu, Ao Shang, Qian-Qian Mao, Hua-Bin Li

**Affiliations:** 1 Guangdong Provincial Key Laboratory of Food, Nutrition and Health, Department of Nutrition, School of Public Health, Sun Yat-Sen University, Guangzhou 510080, China; tanggy5@mail2.sysu.edu.cn (G.-Y.T.); zhaocn@mail2.sysu.edu.cn (C.-N.Z.); xuxy53@mail2.sysu.edu.cn (X.-Y.X.); caoshy3@mail2.sysu.edu.cn (S.-Y.C.); liuq248@mail2.sysu.edu.cn (Q.L.); shangao@mail2.sysu.edu.cn (A.S.); maoqq@mail2.sysu.edu.cn (Q.-Q.M.); 2 Department of Food Science & Technology, School of Agriculture and Biology, Shanghai Jiao Tong University, Shanghai 200240, China

**Keywords:** tea, *Camellia sinensis*, antioxidant activity, polyphenol, catechin, caffeine, theaflavine

## Abstract

Tea has been reported to prevent and manage many chronic diseases, such as cancer, diabetes, obesity, and cardiovascular diseases, and the antioxidant capacity of tea may be responsible for these health benefits. In this study, the antioxidant capacities of fat-soluble, water-soluble, and bound-insoluble fractions of 30 Chinese teas belonging to six categories, namely green, black, oolong, dark, white, and yellow teas, were systematically evaluated, applying ferric-reducing antioxidant power and Trolox equivalent antioxidant capacity assays. In addition, total phenolic contents of teas were determined by Folin–Ciocalteu method, and the contents of 18 main phytochemical compounds in teas were measured by high-performance liquid chromatography (HPLC). The results found that several teas possessed very strong antioxidant capacity, and caffeine, theaflavine, gallic acid, chlorogenic acid, ellagic acid, and kaempferol-3-*O*-glucoside, as well as eight catechins, were the main antioxidant compounds in them. Thus, these teas could be good natural sources of dietary antioxidants, and their extracts might be developed as food additives, nutraceuticals, cosmetics, and pharmaceuticals.

## 1. Introduction

Tea is generally made from the leaves of *Camellia sinensis*, and is a very popular soft drink all over the world. Based on the fermentation degrees in an increasing order, tea can be classified into six categories, including green (unfermented), yellow (slight-fermented), white (mild-fermented), oolong (semi-fermented), black (deep-fermented), and dark (post-fermented) teas [1]. Tea has been widely associated with various health functions, such as the cardiovascular protective, anticancer, antidiabetic, antiobesity, neuroprotective, and hepatoprotective effects [2,3,4,5,6,7,8,9,10]. These beneficial effects can be mainly attributed to the natural antioxidant phytochemicals in tea, especially polyphenols, which may undergo big differences in different teas with diverse genotypes, maturity, producing areas, or fermentation degrees [11,12,13,14,15].

In the present study, the antioxidant capacity of 30 tea samples, which are the best-selling and most commonly consumed teas in China, were systematically evaluated. In addition, their total phenolic contents were determined, and main antioxidant phytochemicals were identified and quantified by HPLC. The results should be helpful for the development of tea-based products, such as food additives, cosmetics, nutraceuticals, and pharmaceuticals.

## 2. Materials and Methods

### 2.1. Chemicals

The 2,2′-azinobis(3-ethylbenothiazoline-6-sulphonic acid) diammonium salt (ABTS), 6-hydroxy-2,5,7,8-tetramethylchromane-2-carboxylic acid (Trolox), 2,4,6-tri(2-pyridyl)-s-triazine (TPTZ), and Folin–Ciocalteu’s phenol reagent were purchased from Sigma-Aldrich (St. Louis, MO, USA). The 18 standard compounds for high-performance liquid chromatography (HPLC) analysis were obtained from Derick Biotechnology Co., Ltd. (Chengdu, China). Tetrahydrofuran, methanol, formic acid, diethyl ether, and ethyl acetate were acquired from Kermel Chemical Factory (Tianjin, China). Acetic acid, sodium acetate, sodium hydroxide, hydrochloric acid, ethylenediaminetetraacetic acid, ascorbic acid, iron(III) chloride hexahydrate, iron(II) sulphate heptahydrate, potassium persulphate, sodium carbonate, ethanol, and n-hexane were bought from Damao Chemical Factory (Tianjin, China). All the reagents were of analytical or HPLC grade, and deionized water was used for all experiments.

### 2.2. Sample Preparation

The 30 common Chinese teas are widely consumed and famous in China. The basic information of these teas is presented in Table 1. The fat-soluble, water-soluble, and bound-insoluble fractions of these teas were acquired using tetrahydrofuran, methanol-acetic acid-water (50:3.7:46.3, *v*/*v*/*v*), and diethyl ether-ethyl acetate (1:1, *v*/*v*) with alkaline digestion according to the procedures described in the literature [16,17,18]. All extracts were preserved at −20 °C before being subjected to relevant tests.

### 2.3. Ferric-Reducing Antioxidant Power (FRAP) Assay

The ferric-reducing antioxidant power (FRAP) assay was conducted according to the method established by Benzie and Strain [19]. FeSO_4_ was used as the standard, and the value was presented as μmol Fe(II)/g dry weight (DW) of the tea.

### 2.4. Trolox Equivalent Antioxidant Capacity (TEAC) Assay

The Trolox equivalent antioxidant capacity (TEAC) assay was performed based on the procedure reported by Re et al. [20]. Trolox was applied as the standard, and the value was displayed as μmol Trolox/g DW of the tea.

### 2.5. Determination of Total Phenolic Content (TPC)

Determination of total phenolic content (TPC) was carried out as described by Singleton et al. [21]. Gallic acid was adopted as the standard, and the value was described as mg gallic acid equivalent (mg GAE)/g DW of the tea.

### 2.6. Detection of Phytochemicals by High-Performance Liquid Chromatography (HPLC)

Caffeine, theaflavine, and polyphenols in the extracts were detected by HPLC based on the literature reported by Cai et al. [22] with small alterations. Briefly, the testing system was comprised of a Waters (Milford, MA, USA) 1525 binary HPLC pump separation module with an auto-injector, a Waters 2996 photodiode array detector (PDAD) and an Agilent Zorbax Extend-C18 column (250 × 4.6 mm, 5 μm, Santa Clara, CA, USA). Gradient elution was performed at 35 °C with the mobile phase composed of methanol (solution A) and 0.1% formic acid solution (solution B), which were routinely delivered at a flow rate of 1.0 mL/min according to the procedure: 0 min, 5% (A); 10 min, 20% (A); 15 min, 22% (A); 20 min, 25% (A); 40 min, 40% (A); 50 min, 42% (A); 60 min, 50% (A); 70 min, 95% (A); 70.10 min, 5% (A); 75 min, 5% (A). A 20 µL of extract sample was injected for HPLC analysis. The spectra were recorded between 200 and 600 nm, and the targeted compounds were identified by retention time and UV-Vis spectra in comparison with the standards and quantified based on the peak area under the maximum absorption wavelength. The value was expressed as mg/g DW of the tea.

### 2.7. Data Analysis

All tests were performed in triplicate, and the values were expressed as mean ± standard deviation (SD). SPSS 22 (International Business Machines Corp, Armonk, NY, USA) and Excel 2007 (Microsoft Corporation, Redmond, WA, USA) were applied for data analysis. One-way ANOVA and post hoc Tukey test were performed to compare means of more than two samples, and *p* value less than 0.05 was defined as statistical significance.

## 3. Results

### 3.1. Ferric-Reducing Antioxidant Power (FRAP) Values of the Tested Teas

FRAP value was used as an important indicator for the antioxidant capacity with regard to reducing ferric ions to ferrous ions, and FRAP results of the 30 teas are displayed in Table 2. The total FRAP values ranged from 611.18 ± 5.09 to 5375.18 ± 228.43 μmol Fe (II)/g DW with a 9-fold difference. Dianqing Tea, Xihu Longjing Tea, Dongting Biluochun Tea, Yongxi Huoqing Tea, and Duyun Maojian Tea exerted the top five reducing capacities, namely 5375.18 ± 228.43, 3926.32 ± 56.00, 3845.21 ± 44.17, 3752.52 ± 96.75, and 3664.97 ± 53.33, μmol Fe(II)/g DW, respectively. Tibetan Tea exhibited the lowest reducing ability of 611.18 ± 5.09 μmol Fe(II)/g DW. In addition, according to the statistical description and non-parametric tests (Table 3), the FRAP values for the three fractions met the following order: water-soluble > bound-insoluble > fat-soluble.

### 3.2. Trolox Equivalent Antioxidant Capacity (TEAC) Values of the Tested Teas

TEAC value was an important index of free radical-scavenging capacity, and the results of the 30 Chinese teas are presented in Table 4. The total TEAC values varied from 326.32 ± 0.48 to 3004.40 ± 112.89 μmol Trolox/g DW with a 9-fold difference. Dianqing Tea, Junshan Yinzhen Tea, Mengding Huangya Tea, Weishan Maojian Tea, and Xihu Longjing Tea showed the top five free radical-scavenging capacities, namely, 3004.40 ± 112.89, 2418.71 ± 26.70, 2303.72 ± 53.67, 2250.40 ± 37.95 and 2125.92 ± 44.43 μmol Trolox/g DW, respectively. Tibetan Tea had the lowest free radical-scavenging capacity of 326.32 ± 0.48 μmol Trolox/g DW. Moreover, according to the statistical description and non-parametric tests (Table 3), TEAC values for the three fractions met the following order: water-soluble > bound-insoluble > fat-soluble bound.

### 3.3. Total Phenolic Content (TPC) of the Tested Teas

TPC was adopted to measure the total contents of phenolic compounds in the 30 Chinese teas, and the results are shown in Table 5. Briefly, the range of total TPC values was 37.25 ± 0.16 to 254.29 ± 15.51 mg GAE/g DW with a 7-fold difference. Dianqing Tea, Xihu Longjing Tea, Junshan Yinzhen Tea, Dongting Biluochun Tea, and Yuan’an Luyuan Tea possessed the top five total phenolic contents, namely 254.29 ± 15.51, 215.39 ± 11.87, 214.72 ± 3.22, 211.20 ± 2.52, and 210.05 ± 7.84 mg GAE/g DW, respectively. Tibetan Tea was observed with the lowest TPC of 37.25 ± 0.16 mg GAE/g DW. In addition, based on the statistical description and non-parametric tests (Table 3), the TPC values for the three fractions met the following order: water-soluble > bound-insoluble > fat-soluble.

### 3.4. Correlations among Ferric-Reducing Antioxidant Power (FRAP), Trolox Equivalent Antioxidant Capacity (TEAC), and Total Phenolic Content (TPC) Values

**Figure 1:**
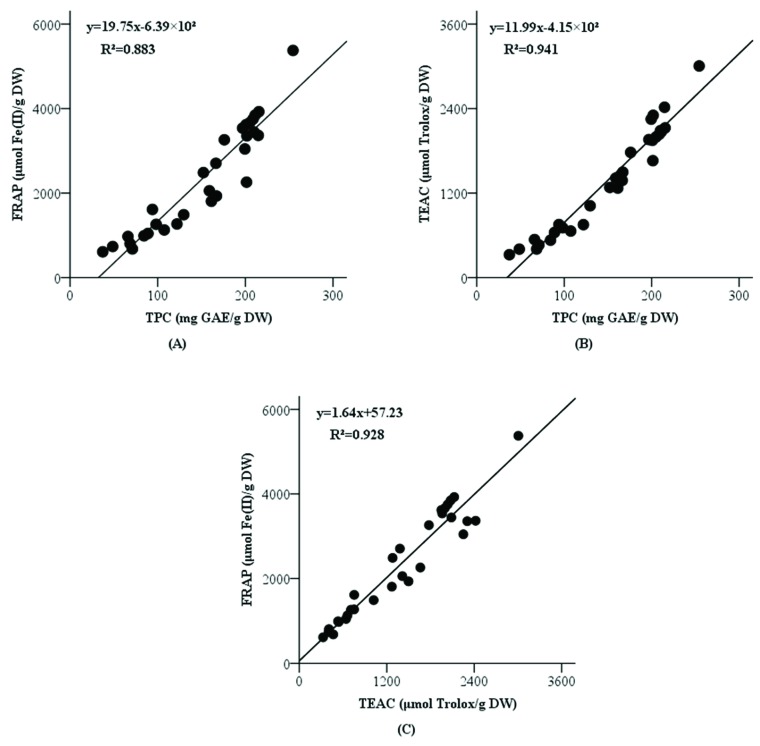
Correlations among FRAP and TPC values (**A**), TEAC and TPC values (**B**), FRAP and TEAC values (**C**). Abbreviations: FRAP, ferric-reducing antioxidant power; TEAC, Trolox equivalent antioxidant capacity; TPC, total phenolic content.

The correlations among FRAP, TEAC, and TPC values (based on the total values of three fractions) were determined by the simple linear regression model, and the results are presented in Figure 1. Both FRAP and TEAC values were significantly and positively correlated with TPC (*R²* = 0.883, *p* < 0.001 and *R²* = 0.941, *p* < 0.001, respectively). These results suggested that the phenolic compounds could be the main components contributing to the antioxidant activities of tea. In addition, FRAP values were positively and remarkably correlated with TEAC values (*R²* = 0.928, *p* < 0.001). Therefore, the antioxidants in tea could possess multiple functions regarding reducing oxidants (like Fe(III)) and scavenging free radicals (like ABTS•^+^).

### 3.5. Systematic Cluster of the Tested Teas

**Figure 2:**
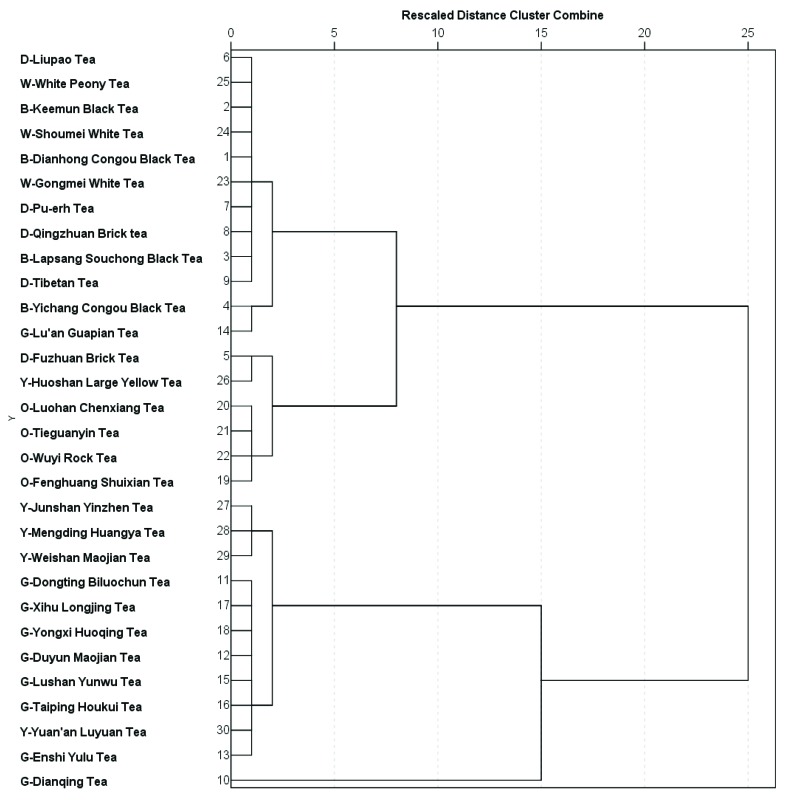
Dendrogram using average linkage (between groups) from systematic cluster analysis for 30 Chinese teas. B, black tea; D, dark tea; G, green tea; O, oolong tea; W, white tea; Y, yellow tea.

Based on the FRAP, TEAC, and TPC values, a systematic cluster analysis for the 30 teas was conducted with cluster numbers from 2 to 6, and the results are summarized in Figure 2. After that, the outcomes of cluster number = 4 were further analyzed using Online Analytical Processing (OLAP) accompanied with variance analysis (ANOVA), and the results are presented in Table 6. In detail, cluster 1 contained 12 teas, which were 4 black teas, 4 dark teas, 3 white teas, and 1 green tea, with the lowest values for FRAP, TEAC, and TPC (1050.03 ± 317.40 μmol Fe(II)/g DW, 600.57 ± 194.85 μmol Trolox/g DW, and 84.66 ± 27.90 mg GAE/g DW, respectively). In addition, cluster 2 comprised all the 4 oolong teas, 1 dark tea, and 1 yellow tea, with relatively low values for FRAP, TEAC, and TPC (2207.42 ± 342.61 μmol Fe(II)/g DW, 1416.88 ± 146.80 μmol Trolox/g DW and 167.83 ± 17.30 mg GAE/g DW, respectively). Moreover, cluster 3 consisted of 7 green teas and 4 yellow teas, with apparently high values for FRAP, TEAC, and TPC (3528.45 ± 265.76 μmol Fe(II)/g DW, 2089.27 ± 180.60 μmol Trolox/g DW, and 203.61 ± 11.11 mg GAE/g DW, respectively). Furthermore, cluster 4 included only 1 tea (green tea), with FRAP, TEAC, and TPC values the highest (5375.18 ± 228.43 μmol Fe(II)/g DW, 3004.40 ± 112.89 μmol Trolox/g DW, and 254.29 ± 15.51 mg GAE/g DW, respectively). Based on the result of ANOVA, all of the differences among the 4 clusters regarding FRAP, TEAC, and TPC values were significant (all *p* < 0.001).

### 3.6. Contents of Phytochemical Compounds in Teas

**Figure 3:**
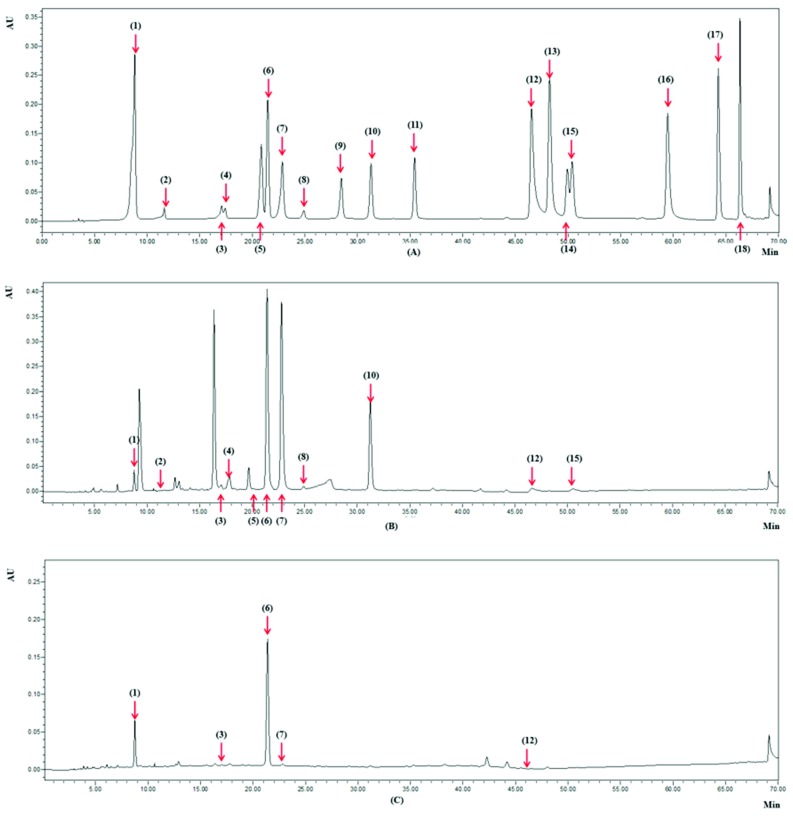
High-performance liquid chromatography (HPLC) Chromatograms under 254 nm of the standard compounds (**A**); Dianqing Tea (**B**); Tibetan Tea (**C**). The numbers in brackets referred to the compounds: gallic acid (**1**); gallocatechin (**2**); epigallocatechin (**3**); catechin (**4**); chlorogenic acid (**5**); caffeine (**6**); epigallocatechin gallate (**7**); epicatechin (**8**); gallocatechin gallate (**9**); epicatechin gallate (**10**); catechin gallate (**11**); ellagic acid (**12**); myricetin (**13**); quercitrin (**14**); kaempferol-3-*O*-glucoside (**15**); quercetin (**16**); theaflavine (**17**); kaempferol (**18**).

Main phytochemicals in 30 Chinese teas, including main catechins (Table 7), caffeine, theaflavine, and other polyphenols (Table 8), were determined by HPLC-PDAD. The chromatograms of the mixed standards and the samples of Dianqing Tea and Tibetan Tea under 254 nm are shown in Figure 3. Eight catechins, including catechin, epicatechin, gallocatechin, epigallocatechin, catechin gallate, epicatechin gallate, gallocatechin gallate, epigallocatechin gallate, four other phenolic compounds, including gallic acid, chlorogenic acid, ellagic acid, and kaempferol-3-*O*-glucoside, caffeine, and theaflavine, were identified in the 30 teas, with epigallocatechin gallate, gallic acid, and caffeine detected and quantified in all Chinese tea samples.

Catechins are the most abundant bioactive compounds in teas. In this study, it was found that epigallocatechin gallate was rich in the tested teas, with a range of 0.539 ± 0.013 to 59.354 ± 1.131 mg/g DW, but the difference was apparently large (up to a 110-fold difference). Green, yellow, and oolong teas were comprised of abundant epigallocatechin, but dark, black, and white teas were not. Dianqing Tea, Yuan’an Luyuan Tea, Xihu Longjing Tea, Yongxi Huoqing Tea, and Junshan Yinzhen Tea contained the top-five contents of epigallocatechin gallate, showing 59.354 ± 1.131, 57.230 ± 0.253, 51.734 ± 0.240, 50.947 ± 0.396, and 50.777 ± 0.224 mg/g DW, respectively. Dianhong Tea, with 0.539 ± 0.013 mg/g DW of epigallocatechin gallate was the lowest one. Additionally, these tea samples, especially green, yellow, and oolong teas, were also detected with remarkably high contents of epigallocatechin (2.288 ± 0.050 to 139.854 ± 1.075 mg/g DW), epicatechin (0.477 ± 0.030 to 13.723 ± 0.216 mg/g DW), and epigallocatechin gallate (0.455 ± 0.037 to 35.395 ± 0.568 mg/g DW). Tieguanyin Tea (139.854 ± 1.075 mg/g DW), Luohan Chenxiang Tea (125.439 ± 0.678 mg/g DW), Lu’an Guapian Tea (100.684 ± 0.561 mg/g DW), Taiping Houkui Tea (74.212 ± 0226 mg/g DW), and Lushan Yunwu Tea (53.447 ± 0.326 mg/g DW) possessed the top-five contents of epigallocatechin. Tieguanyin Tea (13.723 ± 0.216 mg/g DW), Fuzhuan Brick Tea (10.357 ± 0.268 mg/g DW), Weishan Maojian Tea (10.062 ± 0.040 mg/g DW), Duyun Maojian Tea (8.700 ± 0.429 mg/g DW), and Taiping Houkui Tea (8.580 ± 0.211 mg/g DW) contained the top-five contents of epicatechin. Dianqing Tea (35.395 ± 0.568 mg/g DW), Juanshan Yinzhen Tea (30.491 ± 0.101 mg/g DW), Dongting Biluochun Tea (27.893 ± 0.426 mg/g DW), Weishan Maojian Tea (24.710 ± 0.247 mg/g DW), and Mengding Huangya Tea (23.805 ± 0.075 mg/g DW) were shown with the top-five contents of epicatechin gallate.

In addition, for other phytochemical compounds besides catechins in teas, the content of gallic acid was low in all tea samples, ranging from 0.294 ± 0.021 to 3.822 ± 0.111 mg/g DW with a 13-fold difference. Huoshan Large Yellow Tea, Yichang Congou Black Tea, Fenghuang Shuixian Tea, Fuzhuan Brick Tea, and Keemun Black Tea possessed the top-five contents of gallic acid, which were 3.822 ± 0.111, 3.546 ± 0.050, 3.284 ± 0.141, 3.097 ± 0.122, and 2.706 ± 0.117 mg/g DW, respectively. Tieguanyin Tea was shown to have the lowest content of gallic acid, which was 0.294 ± 0.021 mg/g DW. Similarly, the contents of chlorogenic acid, ellagic acid, and kaempferol-3-*O*-glucoside were also relatively low in the tested teas.

**Figure 4:**
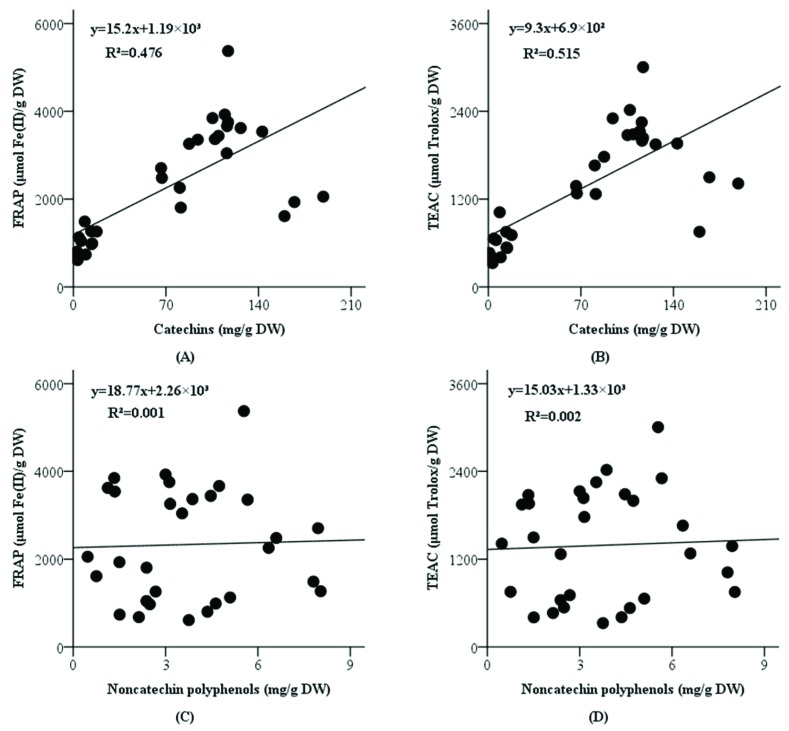
Correlations between FRAP and catechins (**A**), TEAC and catechins (**B**), FRAP and noncatechin polyphenols (**C**) and TEAC and noncatechin polyphenols (**D**). Abbreviations: FRAP, ferric-reducing antioxidant power; TEAC, Trolox equivalent antioxidant capacity.

As polyphenols were suggested as the main antioxidants in teas (Figure 1), we next analyzed the relationships of different polyphenols and antioxidant activities of teas. It was found that the content of catechins had moderate positive correlations (Figure 4A,B) with FRAP values (*R^2^* = 0.476, *p* < 0.001) and TEAC values (*R^2^* = 0.515, *p* < 0.001), while the content of noncatechin polyphenols had no evident linear correlations (Figure 4C,D) with FRAP values (*R^2^* = 0.001, *p* = 0.867) and TEAC values (*R^2^* = 0.002, *p* = 0.819). These results indicate that catechins can be one of the main antioxidants in tea, but noncatechin polyphenols should not be the main contributors.

In addition to this, each tea contained relatively high caffeine content, which varied from 12.273 ± 0.040 to 41.631 ± 0.312 mg/g DW with a small difference (only a 3-fold difference). Yichang Congou Black Tea, Junshan Yinzhen Tea, Yuan’an Luyuan Tea, Dianqing Tea, and Xihu Longjing Tea comprised the top-five content of caffeine, namely 41.631 ± 0.312, 41.457 ± 0.322, 40.737 ± 0.116, 39.764 ± 0.382, and 38.508 ± 0.117 mg/g DW, respectively. The 12.273 ± 0.040 mg/g DW of caffeine in Qingzhuan Brick Tea was the lowest.

Furthermore, Wuyi Rock Tea (oolong tea) and Fuzhuan Brick Tea (dark tea) as well as all of the 4 black teas (Yichang Congou Black Tea, Keemun Black Tea, Dianhong Congou Black Tea, and Lapsang Souchong Black Tea) were found with a spot of theaflavine, and the contents were 0.545 ± 0.011, 0.480 ± 0.008, 0.559 ± 0.018, 0.542 ± 0.010, 0.526 ± 0.019, and 0.488 ± 0.012 mg/g DW, respectively.

## 4. Discussion

### 4.1. Antioxidant Capacities of the Tested Chinese Teas

Many natural products, such as vegetables, fruits, and medicinal plants, possess rich phytochemicals, some of which have been recognized as strong antioxidants [23,24,25,26,27,28,29]. These natural antioxidants are often multifunctional, and their antioxidant capacities can be generally influenced by various factors, e.g., the extraction solvents, extraction conditions, and measurement methods, resulting in the difficulty to completely illustrate the antioxidant capacities only applying a single method [30,31]. In order to maximize the extraction yields of antioxidants from tea, 3 solvents × 2 repeated extraction were adopted in this study [32]. In addition, a reliable antioxidant assessing system demands comprehensive indices, which comprise different experiments to evaluate the antioxidant capacity with diverse mechanisms of action. The FRAP assay was set up based on the power of antioxidants to reduce ferric ions to ferrous ions [19], while the TEAC assay was established on the basis of the capacity of antioxidants to scavenge the ABTS•^+^ free radicals [20]. These two assays are simple, fast, repeatable, and widely used for the evaluation of antioxidant capacity [33,34,35]. In this study, FRAP and TEAC assays were simultaneously used to assess the antioxidant capacities of the 30 Chinese teas.

The FRAP and TEAC values of the tested teas were extremely high compared with other natural products (Table 9). That is, the antioxidant capacities of tea were higher than those of most medicinal plants, edible macro-fungi, vegetables, fruits, fruit wastes (peels and seeds), and wild fruits, as well as edible and wild flowers [33,34,35]. This may be explained by the apparently higher content of phenolic compounds in tea, as revealed in Table 8. Therefore, teas rich in antioxidants may be important natural sources of dietary antioxidants, and their extracts can be used to produce food additives, cosmetics, nutraceuticals, and pharmaceuticals.

Moreover, the FRAP and TEAC values of water-soluble fractions were remarkably higher than those of bound-insoluble fractions, which were mildly higher than those of fat-soluble fractions. These results suggested that the components responsible for the ferric-reducing power and ABTS free radical-scavenging capacity of teas were most water-soluble compounds (approximately 87–93%) with some bound-insoluble (about 5–8%) and fat-soluble (roughly 2–5%) ones.

### 4.2. Antioxidant Phytochemical Components of the Tested Chinese Teas

As demonstrated previously, there were significant and remarkable correlations among FRAP, TEAC, and TPC values. These results suggested that the phenolic compounds could be the major components contributing to the antioxidant capacities of tea, which possessed multiple effects to reduce oxidants and scavenge free radicals. The outcomes demonstrated above were consistent with several previous studies, which have reported that phenolic components were the main contributors responsible for the antioxidant capacities of vegetables, macro-fungi, wild fruits, and flowers [23,37,38,39]. Moreover, many polyphenols have been detected in these natural products, e.g., gallic acid, chlorogenic acid, ferulic acid, anthocyanins, quercetin, rutin, myricetin, and kaempferol glycosides, which exhibited potent antioxidant capacities both in vitro and in vivo [23,37,38,39]. Antioxidant action can be one of the most important mechanisms of the health benefits of these natural products [41,42,43]. As for tea, eight catechins, caffeine, theaflavine, gallic acid, chlorogenic acid, ellagic acid, and kaempferol-3-*O*-glucoside, could be detected. Among them, epicatechin, epigallocatechin, epicatechin gallate, and epigallocatechin gallate were the most abundant polyphenols in tea, especially in the green, yellow, and oolong teas, which generally undergo a low degree of fermentation. Though tea and other natural products contain several common antioxidants, their contents in tea are generally higher.

Tea polyphenols may exert antioxidant capacities through the following mechanisms: (1) straightly reducing oxidants; (2) chelating metal ions; (3) transferring hydrogen; (4) scavenging free radicals; (5) improving activities of antioxidant enzymes; (6) increasing contents of endogenous antioxidants; and (7) regulating antioxidant-related genes [4,44,45,46,47,48,49,50,51]. All of these actions lead to the health functions of tea, such as anticancer, cardiovascular protective, neuroprotective, hepatoprotective, and renoprotective effects [6,52,53,54,55,56]. Thus, several teas rich in antioxidants can be developed into functional foods or nutraceuticals to prevent and treat certain oxidative stress-related chronic diseases.

### 4.3. Comparison of Antioxidant Phytochemicals among Different Chinese Teas

In the light of the outcomes from systematic cluster analysis accompanied by OLAP and ANOVA for cluster number = 4, green tea and yellow tea possessed remarkably high antioxidant capacities and phenolic contents, but Lu’an Guapian Tea (green tea) and Huoshan Large Yellow Tea (yellow tea) were the exceptions. In addition, oolong tea was in the middle position. Meanwhile, white tea, black tea, and dark tea exerted relatively low antioxidant capacities and phenolic contents. Thus, fermentation degree can be a crucial factor that influences the antioxidant capacity and phenolic content of tea. Tea undergoing higher fermentation degree might have lower antioxidant capacity and phenolic content, since tea polyphenols, especially catechins, may oxidize and polymerize during fermentation, generating complicated tea pigments like theaflavins, thearubigins, and theabrownins [57,58,59]. Moreover, the maturity of tea leaves should also be taken into consideration, because the antioxidant capacity and phenolic content would decrease accompanied with the increase of tea leaf maturity [60], which may partially explain why white tea (made of old tea leaves) exhibited relatively low antioxidant capacity and phenolic content, although it has a low fermentation degree. On the other hand, it was reported that the bioavailability of fermented tea using microbes, such as bacteria, yeasts, and fungi, could be significantly higher compared to unfermented tea [61,62]. For example, green and black teas have been observed to improve endothelial function with equal effectiveness, although green tea possesses higher antioxidant activity and phenolic content, it has a lower bioavailability [63].

## 5. Conclusions

In conclusion, teas here studied possessed remarkably high antioxidant capacities regarding ferric-reducing and free radical-scavenging capacities. In addition, eight catechins, caffeine, theaflavine, and several other phenolic compounds, including gallic acid, chlorogenic acid, ellagic acid, and kaempferol-3-*O*-glucoside, were detected in these Chinese teas. Compared with dark, black, and white teas, green, yellow, and oolong teas exerted stronger antioxidant capacity and contained more polyphenols, especially catechins like epicatechin, epigallocatechin, epicatechin gallate, and epigallocatechin gallate. Overall, tea is a good natural source of dietary antioxidant phytochemicals, and can be used to produce food additives, functional foods, nutraceuticals, and cosmetics.

## Figures and Tables

**Table 1:** Basic information about the 30 Chinese teas.

No.	Name	Category	Fermentation Degree	Production Place
1	Dianhong Congou Black Tea	Black tea	Deep-fermented	Kunming, Yunnan
2	Keemun Black Tea	Black tea	Deep-fermented	Qimen, Anhui
3	Lapsang Souchong Black Tea	Black tea	Deep-fermented	Wuyishan, Fujian
4	Yichang Congou Black Tea	Black tea	Deep-fermented	Yichang, Hubei
5	Fuzhuan Brick Tea	Dark tea	Post-fermented	Anhua, Hubei
6	Liupao Tea	Dark tea	Post-fermented	Wuzhou, Guangxi
7	Pu-erh Tea	Dark tea	Post-fermented	Pu’er, Yunnan
8	Qingzhuan Brick tea	Dark tea	Post-fermented	Chibi, Hubei
9	Tibetan Tea	Dark tea	Post-fermented	Ya’an, Sichuan
10	Dianqing Tea	Green tea	Unfermented	Kunming, Yunnan
11	Dongting Biluochun Tea	Green tea	Unfermented	Suzhou, Jiangsu
12	Duyun Maojian Tea	Green tea	Unfermented	Duyun, Guizhou
13	Enshi Yulu Tea	Green tea	Unfermented	Enshi, Hubei
14	Lu’an Guapian Tea	Green tea	Unfermented	Lu’an, Anhui
15	Lushan Yunwu Tea	Green tea	Unfermented	Jiujiang, Jiangxi
16	Taiping Houkui Tea	Green tea	Unfermented	Huangshan, Anhui
17	Xihu Longjing Tea	Green tea	Unfermented	Hangzhou, Zhejiang
18	Yongxi Huoqing Tea	Green tea	Unfermented	Jingxian, Anhui
19	Fenghuang Shuixian Tea	Oolong tea	Semi-fermented	Chao’an, Guangdong
20	Luohan Chenxiang Tea	Oolong tea	Semi-fermented	Mengdingshan, Sichuan
21	Tieguanyin Tea	Oolong tea	Semi-fermented	Anxi, Fujian
22	Wuyi Rock Tea	Oolong tea	Semi-fermented	Wuyishan, Fujian
23	Gongmei White Tea	White tea	Mild-fermented	Nanping, Fujian
24	Shoumei White Tea	White tea	Mild-fermented	Nanping, Fujian
25	White Peony Tea	White tea	Mild-fermented	Nanping, Fujian
26	Huoshan Large Yellow Tea	Yellow tea	Light-fermented	Lu’an, Anhui
27	Junshan Yinzhen Tea	Yellow tea	Light-fermented	Yueyang, Hunan
28	Mengding Huangya Tea	Yellow tea	Light-fermented	Mengdingshan, Sichuan
29	Weishan Maojian Tea	Yellow tea	Light-fermented	Ningxiang, Hunan
30	Yuan’an Luyuan Tea	Yellow tea	Light-fermented	Yichang, Hubei

**Table 2:** Ferric-reducing antioxidant power (FRAP) values of the 30 common Chinese teas.

No.	Name	Category	FRAP Value (μmol Fe(II)/g DW)				
			Fat-Soluble Fraction	Water-Soluble Fraction	Bound-Insoluble Fraction	Total	Mean ± SD of Categories
1	Dianhong Congou Black Tea	Black tea	11.53 ± 0.27	1142.49 ± 5.55	115.29 ± 0.54	1269.31 ± 5.88	1141.58 ± 13.92 ^a^
2	Keemun Black Tea	Black tea	5.20 ± 0.17	1034.93 ± 36.25	88.41 ± 1.93	1128.53 ± 34.52	
3	Lapsang Souchong Black Tea	Black tea	15.53 ± 0.58	585.60 ± 7.42	78.32 ± 2.79	679.45 ± 8.29	
4	Yichang Congou Black Tea	Black tea	4.56 ± 0.43	1371.38 ± 5.05	113.07 ± 1.53	1489.01 ± 6.98	
5	Fuzhuan Brick Tea	Dark tea	65.53 ± 1.89	2318.76 ± 77.12	100.66 ± 3.01	2484.94 ± 80.20	1124.96 ± 23.87 ^a^
6	Liupao Tea	Dark tea	5.01 ± 0.29	922.49 ± 12.03	62.13 ± 0.70	989.62 ± 11.40	
7	Pu-erh Tea	Dark tea	5.10 ± 0.11	725.60 ± 18.04	72.77 ± 1.16	803.46 ± 16.99	
8	Qingzhuan Brick tea	Dark tea	38.20 ± 0.73	648.71 ± 6.16	48.68 ± 0.96	735.59 ± 5.64	
9	Tibetan Tea	Dark tea	31.31 ± 0.69	534.04 ± 4.68	45.82 ± 0.59	611.18 ± 5.09	
10	Dianqing Tea	Green tea	250.73 ± 8.97	4937.24 ± 236.20	187.20 ± 0.88	5375.18 ± 228.43	3621.75 ± 81.44 ^b^
11	Dongting Biluochun Tea	Green tea	230.51 ± 10.31	3515.73 ± 37.33	98.96 ± 1.56	3845.21 ± 44.17	
12	Duyun Maojian Tea	Green tea	71.31 ± 1.58	3442.84 ± 50.69	150.81 ± 4.30	3664.97 ± 53.33	
13	Enshi Yulu Tea	Green tea	44.26 ± 1.55	3037.51 ± 13.42	179.64 ± 2.28	3261.41 ± 14.50	
14	Lu’an Guapian Tea	Green tea	67.92 ± 1.01	1413.60 ± 43.90	131.76 ± 3.17	1613.28 ± 43.98	
15	Lushan Yunwu Tea	Green tea	93.42 ± 2.68	3396.62 ± 83.02	128.98 ± 3.08	3619.02 ± 83.57	
16	Taiping Houkui Tea	Green tea	139.26 ± 4.23	3272.18 ± 117.50	126.42 ± 2.34	3537.86 ± 112.24	
17	Xihu Longjing Tea	Green tea	196.62 ± 4.99	3650.84 ± 53.42	78.85 ± 5.71	3926.32 ± 56.00	
18	Yongxi Huoqing Tea	Green tea	90.20 ± 1.69	3512.18 ± 96.79	150.14 ± 1.39	3752.52 ± 96.75	
19	Fenghuang Shuixian Tea	Oolong tea	43.40 ± 4.37	2069.87 ± 55.49	144.26 ± 1.51	2257.52 ± 50.42	2013.37 ± 26.17 ^a^
20	Luohan Chenxiang Tea	Oolong tea	26.03 ± 0.29	1827.20 ± 12.22	79.77 ± 0.30	1933.00 ± 12.52	
21	Tieguanyin Tea	Oolong tea	84.73 ± 5.69	1911.64 ± 10.10	59.82 ± 0.39	2056.20 ± 4.96	
22	Wuyi Rock Tea	Oolong tea	20.62 ± 1.40	1696.53 ± 34.87	89.60 ± 1.81	1806.75 ± 36.77	
23	Gongmei White Tea	White tea	31.03 ± 1.69	1149.16 ± 16.67	81.63 ± 1.86	1261.82 ± 18.31	1093.64 ± 16.82 ^a^
24	Shoumei White Tea	White tea	28.09 ± 1.25	934.49 ± 28.98	82.85 ± 1.39	1045.43 ± 29.22	
25	White Peony Tea	White tea	32.37 ± 0.76	849.16 ± 2.04	92.16 ± 1.47	973.68 ± 2.92	
26	Huoshan Large Yellow Tea	Yellow tea	77.64 ± 2.28	2539.20 ± 14.11	89.24 ± 0.50	2706.08 ± 15.16	3182.34 ± 31.31 ^b^
27	Junshan Yinzhen Tea	Yellow tea	144.81 ± 3.73	3137.42 ± 26.71	84.27 ± 1.97	3366.50 ± 21.49	
28	Mengding Huangya Tea	Yellow tea	112.03 ± 5.95	3173.87 ± 16.22	68.02 ± 1.80	3353.92 ± 10.48	
29	Weishan Maojian Tea	Yellow tea	82.53 ± 4.64	2862.76 ± 80.24	98.46 ± 1.17	3043.75 ± 78.43	
30	Yuan’an Luyuan Tea	Yellow tea	93.31 ± 3.08	3254.40 ± 29.70	93.74 ± 0.19	3441.45 ± 30.99	

Abbreviations: FRAP, ferric-reducing antioxidant power; DW, dry weight; SD, standard deviation. Different superscript lowercase letters (^a,b^) indicated statistical significance (*p* < 0.05).

**Table 3:** Comparison among FRAP, TEAC, and TPC of different tea fractions.

Index	Fraction	Statistical Description					*p* Value by Non-Parametric Test				
		MIN	Q_L_	M	Q_U_	MAX	Item	M-W	Moses	K-S	W-W
FRAP	1	4.56	24.68	54.89	93.34	250.73	a	<0.001	<0.001	<0.001	<0.001
	2	534.04	1009.82	1990.76	3258.84	4937.24	b	<0.001	<0.001	<0.001	<0.001
	3	45.82	78.72	90.88	127.06	187.20	c	=0.003	=1.000	=0.001	=0.025
TEAC	1	9.25	24.17	45.53	66.21	137.97	a	<0.001	<0.001	<0.001	<0.001
	2	277.41	568.63	1295.08	1881.65	2754.98	b	<0.001	<0.001	<0.001	<0.001
	3	34.34	51.52	61.42	77.67	111.45	c	=0.005	=1.000	=0.016	0.181
TPC	1	1.56	3.47	4.75	6.50	11.26	a	<0.001	<0.001	<0.001	<0.001
	2	32.26	81.37	149.58	190.77	236.50	b	<0.001	<0.001	<0.001	<0.001
	3	3.08	5.36	6.43	8.45	11.04	c	=0.006	=1.000	=0.016	=0.347

Note: 1, fat-soluble fraction; 2, water-soluble fraction; 3, bound-insoluble fraction; a, non-parametric test between 1 and 2; b, non-parametric test between 2 and 3; c, non-parametric test between 1 and 3; FRAP, ferric-reducing antioxidant power; K-S, Kolmogorov-Smirnov Test; M, median; MAX, the maximum value; MIN, the minimum value; Moses, Moses Test; M-W, Mann-Whitney test; Q_L_, the lower quartile; Q_U_, the upper quartile; TEAC, Trolox equivalent antioxidant capacity; TPC, total phenolic content; W-W, Wald-Wolfowitz Test.

**Table 4:** Trolox equivalent antioxidant capacity (TEAC) values of the 30 common Chinese teas.

No.	Name	Category	TEAC Value (μmol Trolox/g DW)				
			Fat-Soluble Fraction	Water-Soluble Fraction	Bound-Insoluble Fraction	Total	Mean ± SD of Categories
1	Dianhong Congou Black Tea	Black tea	44.84 ± 1.38	627.92 ± 6.80	78.68 ± 1.19	751.44 ± 7.20	724.28 ± 12.63 ^a^
2	Keemun Black Tea	Black tea	26.43 ± 0.70	570.24 ± 17.10	64.24 ± 1.54	660.91 ± 15.26	
3	Lapsang Souchong Black Tea	Black tea	53.14 ± 2.44	355.48 ± 8.80	56.04 ± 1.92	464.67 ± 8.89	
4	Yichang Congou Black Tea	Black tea	24.12 ± 0.77	918.66 ± 18.82	77.34 ± 1.26	1020.11 ± 19.17	
5	Fuzhuan Brick Tea	Dark tea	40.58 ± 0.66	1172.97 ± 21.52	66.19 ± 1.19	1279.74 ± 21.17	589.43 ± 8.35 ^a^
6	Liupao Tea	Dark tea	22.90 ± 1.29	460.32 ± 9.97	48.23 ± 1.04	531.45 ± 9.97	
7	Pu-erh Tea	Dark tea	19.45 ± 1.11	338.44 ± 5.76	48.13 ± 1.11	406.02 ± 6.98	
8	Qingzhuan Brick tea	Dark tea	17.85 ± 1.19	351.43 ± 3.59	34.34 ± 0.28	403.62 ± 3.15	
9	Tibetan Tea	Dark tea	9.25 ± 0.40	277.41 ± 0.00	39.66 ± 0.45	326.32 ± 0.48	
10	Dianqing Tea	Green tea	137.97 ± 3.13	2754.98 ± 113.83	111.45 ± 2.50	3004.40 ± 112.89	1964.50 ± 38.29 ^b^
11	Dongting Biluochun Tea	Green tea	127.03 ± 3.43	1883.90 ± 11.31	64.02 ± 0.88	2074.94 ± 9.06	
12	Duyun Maojian Tea	Green tea	41.05 ± 1.48	1853.93 ± 27.46	102.89 ± 1.82	1997.86 ± 25.04	
13	Enshi Yulu Tea	Green tea	29.42 ± 0.49	1639.68 ± 27.34	109.31 ± 4.09	1778.42 ± 30.71	
14	Lu’an Guapian Tea	Green tea	42.57 ± 0.31	635.75 ± 11.53	75.13 ± 0.34	753.45 ± 11.73	
15	Lushan Yunwu Tea	Green tea	47.95 ± 0.54	1820.97 ± 44.12	79.77 ± 1.11	1948.69 ± 45.05	
16	Taiping Houkui Tea	Green tea	74.28 ± 0.43	1808.98 ± 26.34	77.21 ± 0.77	1960.47 ± 26.50	
17	Xihu Longjing Tea	Green tea	117.50 ± 4.53	1955.81 ± 40.78	52.61 ± 0.44	2125.92 ± 44.43	
18	Yongxi Huoqing Tea	Green tea	48.85 ± 1.35	1880.90 ± 38.23	106.61 ± 2.24	2036.36 ± 39.18	
19	Fenghuang Shuixian Tea	Oolong tea	38.72 ± 2.22	1541.83 ± 16.35	80.07 ± 0.70	1660.62 ± 18.36	1460.46 ± 22.23 ^b^
20	Luohan Chenxiang Tea	Oolong tea	73.86 ± 2.98	1376.32 ± 22.44	47.53 ± 4.07	1497.72 ± 23.35	
21	Tieguanyin Tea	Oolong tea	61.30 ± 1.59	1311.56 ± 35.34	40.75 ± 1.41	1413.61 ± 33.12	
22	Wuyi Rock Tea	Oolong tea	66.52 ± 2.19	1141.73 ± 17.45	61.64 ± 1.19	1269.89 ± 14.09	
23	Gongmei White Tea	White tea	21.85 ± 0.50	628.51 ± 21.09	57.27 ± 0.21	707.63 ± 20.78	629.61 ± 10.92 ^a^
24	Shoumei White Tea	White tea	20.03 ± 1.33	563.80 ± 7.43	57.18 ± 1.13	641.01 ± 7.33	
25	White Peony Tea	White tea	24.18 ± 0.23	452.93 ± 4.48	63.08 ± 0.65	540.19 ± 4.64	
26	Huoshan Large Yellow Tea	Yellow tea	46.21 ± 1.06	1278.60 ± 27.90	54.92 ± 2.72	1379.72 ± 29.46	2087.81 ± 42.89 ^b^
27	Junshan Yinzhen Tea	Yellow tea	80.05 ± 0.63	2278.99 ± 27.87	59.67 ± 1.68	2418.71 ± 26.70	
28	Mengding Huangya Tea	Yellow tea	66.10 ± 1.29	2192.70 ± 53.25	44.92 ± 1.04	2303.72 ± 53.67	
29	Weishan Maojian Tea	Yellow tea	50.07 ± 1.43	2139.15 ± 38.99	61.19 ± 2.41	2250.40 ± 37.95	
30	Yuan’an Luyuan Tea	Yellow tea	56.30 ± 0.50	1971.03 ± 64.83	59.16 ± 1.42	2086.49 ± 66.69	

Abbreviations: DW, dry weight; SD, standard deviation; TEAC, Trolox equivalent antioxidant capacity. Different superscript lowercase letters (^a,b^) indicated statistical significance (*p* < 0.05).

**Table 5:** Total phenolic content (TPC) values of the 30 common Chinese teas.

No.	Name	Category	TPC Value (mg GAE/g DW)				
			Fat-Soluble Fraction	Water-Soluble Fraction	Bound-Insoluble Fraction	Total	Mean ± SD of Categories
1	Dianhong Congou Black Tea	Black tea	6.43 ± 0.55	106.68 ± 1.26	8.86 ± 0.06	121.97 ± 1.31	107.54 ± 1.80 ^a^
2	Keemun Black Tea	Black tea	4.72 ± 0.08	96.26 ± 3.42	6.47 ± 0.03	107.44 ± 3.43	
3	Lapsang Souchong Black Tea	Black tea	2.31 ± 0.05	62.51 ± 1.65	6.29 ± 0.10	71.11 ± 1.63	
4	Yichang Congou Black Tea	Black tea	3.86 ± 0.23	117.54 ± 0.84	8.25 ± 0.01	129.64 ± 0.85	
5	Fuzhuan Brick Tea	Dark tea	4.21 ± 0.18	141.06 ± 3.39	6.89 ± 0.07	152.17 ± 3.57	78.16 ± 1.33 ^a^
6	Liupao Tea	Dark tea	4.31 ± 0.13	74.28 ± 1.38	5.74 ± 0.06	84.33 ± 1.37	
7	Pu-erh Tea	Dark tea	4.10 ± 0.10	59.52 ± 0.72	4.79 ± 0.05	68.40 ± 0.81	
8	Qingzhuan Brick tea	Dark tea	2.37 ± 0.06	42.95 ± 0.77	3.34 ± 0.05	48.66 ± 0.75	
9	Tibetan Tea	Dark tea	1.91 ± 0.07	32.26 ± 0.20	3.08 ± 0.12	37.25 ± 0.16	
10	Dianqing Tea	Green tea	7.23 ± 0.22	236.50 ± 15.52	10.56 ± 0.21	254.29 ± 15.51	195.79 ± 5.45 ^b^
11	Dongting Biluochun Tea	Green tea	6.66 ± 0.20	198.52 ± 2.45	6.02 ± 0.11	211.20 ± 2.52	
12	Duyun Maojian Tea	Green tea	4.12 ± 0.04	191.19 ± 2.53	9.71 ± 0.23	205.02 ±2.74	
13	Enshi Yulu Tea	Green tea	2.34 ± 0.16	162.53 ± 3.00	11.04 ± 0.87	175.91 ± 2.01	
14	Lu’an Guapian Tea	Green tea	4.03 ± 0.05	81.52 ± 2.34	8.41 ± 0.18	93.96 ± 2.47	
15	Lushan Yunwu Tea	Green tea	5.46 ± 0.11	186.77 ± 4.90	8.56 ± 0.19	200.80 ± 4.76	
16	Taiping Houkui Tea	Green tea	8.65 ± 0.06	179.88 ± 1.80	8.21 ± 0.18	196.74 ± 1.82	
17	Xihu Longjing Tea	Green tea	5.90 ± 0.29	204.88 ± 11.38	4.61 ± 0.20	215.39 ± 11.87	
18	Yongxi Huoqing Tea	Green tea	5.40 ± 0.02	193.67 ± 5.26	9.75 ± 0.11	208.83 ± 5.32	
19	Fenghuang Shuixian Tea	Oolong tea	3.84 ± 0.31	188.80 ± 2.08	8.73 ± 0.28	201.36 ± 1.74	172.11 ± 2.09 ^b^
20	Luohan Chenxiang Tea	Oolong tea	11.26 ± 0.54	150.49 ± 1.77	5.19 ± 0.10	166.94 ± 1.64	
21	Tieguanyin Tea	Oolong tea	6.44 ± 0.51	148.66 ± 1.82	3.81 ± 0.08	158.91 ± 2.17	
22	Wuyi Rock Tea	Oolong tea	10.67 ± 0.20	144.46 ± 2.89	6.10 ± 0.05	161.23 ± 2.83	
23	Gongmei White Tea	White tea	1.57 ± 0.04	90.11 ± 0.57	6.59 ± 0.37	98.28 ± 0.92	84.39 ± 0.79 ^a^
24	Shoumei White Tea	White tea	1.56 ± 0.08	80.90 ± 0.81	6.40 ± 0.07	88.87 ± 0.93	
25	White Peony Tea	White tea	1.73 ± 0.04	57.25 ± 0.45	7.06 ± 0.07	66.04 ± 0.51	
26	Huoshan Large Yellow Tea	Yellow tea	4.79 ± 0.17	156.15 ± 1.96	5.42 ± 0.10	166.35 ± 1.93	198.44 ± 5.39 ^b^
27	Junshan Yinzhen Tea	Yellow tea	9.35 ± 0.16	199.41 ± 3.27	5.97 ± 0.04	214.72 ± 3.22	
28	Mengding Huangya Tea	Yellow tea	6.82 ± 0.15	190.63 ± 3.98	4.22 ± 0.15	201.67 ± 3.82	
29	Weishan Maojian Tea	Yellow tea	4.83 ± 0.28	187.72 ± 10.14	6.87 ± 0.01	199.42 ± 10.13	
30	Yuan’an Luyuan Tea	Yellow tea	5.56 ± 0.10	198.52 ± 7.76	5.97 ± 0.08	210.05 ± 7.84	

Abbreviations: DW, dry weight; GAE, gallic acid equivalent; SD, standard deviation; TPC, total phenolic content. Different superscript lowercase letters (^a,b^) indicated statistical significance (*p* < 0.05).

**Table 6:** Online Analytical Processing (OLAP) Cube based on systematic cluster analysis for 30 Chinese teas (cluster number = 4).

Average Linkage		FRAP	TEAC	TPC
1	SUM	12600.37	7206.81	1015.94
	N	12	12	12
	Mean	1050.03	600.57	84.66
	SD	317.40	194.85	27.90
	SUM/SUMT (%)	18.0%	17.3%	22.5%
	N/NT (%)	40.0%	40.0%	40.0%
2	SUM	13244.50	8501.30	1006.96
	N	6	6	6
	Mean	2207.42	1416.88	167.83
	SD	342.61	146.80	17.30
	SUM/SUMT (%)	18.9%	20.4%	22.3%
	N/NT (%)	20.0%	20.0%	20.0%
3	SUM	38812.92	2298200	2239.75
	N	11	11	11
	Mean	3528.45	2089.27	203.61
	SD	265.76	180.60	11.11
	SUM/SUMT (%)	55.4%	55.1%	49.6%
	N/NT (%)	36.7%	36.7%	36.7%
4	SUM	5375.18	3004.40	254.29
	N	1	1	1
	Mean	5375.18	3004.40	254.29
	SD	NA	NA	NA
	SUM/SUMT (%)	7.7%	7.2%	5.6%
	N/NT (%)	3.3%	3.3%	3.3%
Total	SUM	70032.96	41694.52	4516.94
	N	30	30	30
	Mean	2334.43	1389.82	150.57
	SD	1276.08	750.16	60.72

Abbreviations: FRAP, ferric-reducing antioxidant power; TEAC, Trolox equivalent antioxidant capacity; TPC, total phenolic content.

**Table 7:** The contents (mg/g DW) of catechins in 30 Chinese teas.

No.	Name	Category	Catechin	Epicatechin	Gallocatechin	Epigallocatechin	Catechin Gallate	Epicatechin Gallate	Gallocatechin Gallate	Epigallocatechin Gallate
1	Dianhong Congou Black Tea	Black Tea	-	0.796 ± 0.047	1.098 ± 0.052	8.479 ± 0.500	-	2.583 ± 0.077	-	0.539 ± 0.013
2	Keemun Black Tea	Black Tea	-	0.477 ± 0.030	-	-	-	1.499 ± 0.033	-	2.164 ± 0.102
3	Lapsang Souchong Black Tea	Black Tea	-	-	-	-	-	-	-	0.761 ± 0.031
4	Yichang Congou Black Tea	Black Tea	-	0.740 ± 0.033	-	-	-	3.511 ± 0.070	0.510 ± 0.013	3.795 ± 0.089
5	Fuzhuan Brick Tea	Dark Tea	4.930 ± 0.240	10.357 ± 0.268	5.535 ± 0.128	23.430 ± 0.375	-	10.881 ± 0.105	0.933 ± 0.063	10.885 ± 0.259
6	Liupao Tea	Dark Tea	1.667 ± 0.063	3.886 ± 0.112	2.120 ± 0.150	5.440 ± 0.171	-	0.455 ± 0.037	-	0.647 ± 0.015
7	Pu-erh Tea	Dark Tea	-	1.574 ± 0.086	-	-	-	0.515 ± 0.040	-	0.584 ± 0.044
8	Qingzhuan Brick tea	Dark Tea	-	0.977 ± 0.056	1.062 ± 0.049	5.200 ± 0.140	-	0.542 ± 0.015	-	1.479 ± 0.083
9	Tibetan Tea	Dark Tea	-	-	-	2.288 ± 0.050	-	-	-	0.886 ± 0.027
10	Dianqing Tea	Green Tea	1.315 ± 0.084	5.970 ± 0.210	1.864 ± 0.080	13.094 ± 0.256	-	35.395 ± 0.568	-	59.354 ± 1.131
11	Dongting Biluochun Tea	Green Tea	0.988 ± 0.039	6.310 ± 0.272	1.824 ± 0.051	24.522 ± 0.060	-	27.893 ± 0.426	0.630 ± 0.026	43.070 ± 0.209
12	Duyun Maojian Tea	Green Tea	-	8.700 ± 0.429	2.814 ± 0.167	42.063 ± 0.126	-	18.443 ± 0.537	1.137 ± 0.062	43.056 ± 0.455
13	Enshi Yulu Tea	Green Tea	-	6.443 ± 0.166	2.135 ± 0.140	29.070 ± 0.484	-	16.774 ± 0.090	-	33.102 ± 0.594
14	Lu’an Guapian Tea	Green Tea	-	7.352 ± 0.147	3.015 ± 0.121	100.684 ± 0.561	-	7.599 ± 0.119	0.842 ± 0.044	40.161 ± 0.887
15	Lushan Yunwu Tea	Green Tea	-	6.377 ± 0.150	3.277 ± 0.150	53.447 ± 0.326	-	15.130 ± 0.431	-	48.272 ± 0.363
16	Taiping Houkui Tea	Green Tea	-	8.580 ± 0.211	3.121 ± 0.092	74.212 ± 0.226	-	11.264 ± 0.097	0.640 ± 0.020	45.016 ± 0.222
17	Xihu Longjing Tea	Green Tea	-	5.380 ± 0.216	4.002 ± 0.112	24.494 ± 0.467	0.645 ± 0.036	22.364 ± 0.869	5.844 ± 0.173	51.734 ± 0.240
18	Yongxi Huoqing Tea	Green Tea	-	6.260 ± 0.303	2.630 ± 0.165	38.486 ± 0.994	-	18.064 ± 0.181	0.601 ± 0.019	50.947 ± 0.396
19	Fenghuang Shuixian Tea	Oolong Tea	-	1.579 ± 0.089	2.509 ± 0.121	31.253 ± 0.206	-	8.435 ± 0.270	-	36.704 ± 0.362
20	Luohan Chenxiang Tea	Oolong Tea	-	7.531 ± 0.017	8.088 ± 0.092	125.439 ± 0.678	-	3.683 ± 0.102	-	22.396 ± 0.505
21	Tieguanyin Tea	Oolong Tea	0.775 ± 0.052	13.723 ± 0.216	3.938 ± 0.146	139.854 ± 1.075	-	6.471 ± 0.235	0.562 ± 0.022	23.663 ± 0.308
22	Wuyi Rock Tea	Oolong Tea	-	4.337 ± 0.223	11.528 ± 0.079	36.826 ± 0.668	0.981 ± 0.121	5.083 ± 0.122	2.261 ± 0.111	20.211 ± 0.223
23	Gongmei White Tea	White Tea	-	-	-	8.419 ± 0.143	-	3.144 ± 0.123	-	6.010 ± 0.083
24	Shoumei White Tea	White Tea	-	-	-	-	-	2.270 ± 0.062	-	3.537 ± 0.072
25	White Peony Tea	White Tea	-	1.311 ± 0.033	-	-	-	3.841 ± 0.125	-	8.539 ± 0.169
26	Huoshan Large Yellow Tea	Yellow Tea	2.040 ± 0.054	2.956 ± 0.115	11.858 ± 0.039	14.340 ± 0.135	1.608 ± 0.026	5.549 ± 0.059	10.787 ± 0.108	17.209 ± 0.177
27	Junshan Yinzhen Tea	Yellow Tea	1.366 ± 0.043	6.196 ± 0.178	2.736 ± 0.102	13.661 ± 0.196	0.351 ± 0.014	30.491 ± 0.101	1.447 ± 0.066	50.777 ± 0.224
28	Mengding Huangya Tea	Yellow Tea	-	0.968 ± 0.056	4.844 ± 0.064	22.950 ± 0.102	-	23.805 ± 0.075	2.361 ± 0.128	39.125 ± 0.082
29	Weishan Maojian Tea	Yellow Tea	-	10.062 ± 0.040	2.818 ± 0.072	45.484 ± 0.057	-	-	24.710 ± 0.247	32.856 ± 0.060
30	Yuan’an Luyuan Tea	Yellow Tea	-	5.959 ± 0.147	3.918 ± 0.051	19.877 ± 0.176	-	21.373 ± 0.027	1.388 ± 0.043	57.230 ± 0.253

DW, dry weight, “-“ means not detected.

**Table 8:** The contents (mg/g DW) of other main phytochemicals besides catechins in 30 Chinese teas.

No.	Name	Category	Gallic Acid	Chlorogenic Acid	Caffeine	Ellagic Acid	Kaempferol-3-*O*-Glucoside	Theaflavine
1	Dianhong Congou Black Tea	Black Tea	2.693 ± 0.161	0.187 ± 0.005	35.283 ± 0.340	3.572 ± 0.087	1.588 ± 0.046	0.526 ± 0.019
2	Keemun Black Tea	Black Tea	2.706 ± 0.117	0.176 ± 0.005	31.452 ± 0.140	2.214 ± 0.070	-	0.542 ± 0.010
3	Lapsang Souchong Black Tea	Black Tea	1.748 ± 0.050	-	23.759 ± 0.150	-	0.385 ± 0.027	0.488 ± 0.012
4	Yichang Congou Black Tea	Black Tea	3.546 ± 0.050	0.188 ± 0.005	41.631 ± 0.312	2.614 ± 0.078	1.454 ± 0.099	0.559 ± 0.018
5	Fuzhuan Brick Tea	Dark Tea	3.097 ± 0.122	0.284 ± 0.016	27.075 ± 0.166	2.213 ± 0.067	1.002 ± 0.040	0.480 ± 0.008
6	Liupao Tea	Dark Tea	2.003 ± 0.018	-	30.565 ± 0.162	2.108 ± 0.022	0.519 ± 0.016	-
7	Pu-erh Tea	Dark Tea	1.644 ± 0.081	-	31.320 ± 0.310	2.165 ± 0.072	0.550 ± 0.043	-
8	Qingzhuan Brick tea	Dark Tea	1.507 ± 0.031	-	12.273 ± 0.040	-	-	-
9	Tibetan Tea	Dark Tea	2.203 ± 0.062	-	16.930 ± 0.101	1.553 ± 0.003	-	-
10	Dianqing Tea	Green Tea	1.430 ± 0.086	0.374 ± 0.016	39.764 ± 0.382	2.135 ± 0.037	1.605 ± 0.067	-
11	Dongting Biluochun Tea	Green Tea	0.708 ± 0.046	0.190 ± 0.006	31.993 ± 0.551	-	0.434 ± 0.030	-
12	Duyun Maojian Tea	Green Tea	1.129 ± 0.071	-	36.230 ± 0.563	1.875 ± 0.017	1.737 ± 0.090	-
13	Enshi Yulu Tea	Green Tea	1.392 ± 0.079	-	34.706 ± 0.383	1.756 ± 0.025	-	-
14	Lu’an Guapian Tea	Green Tea	0.533 ± 0.032	0.218 ± 0.009	29.232 ± 0.438	-	-	-
15	Lushan Yunwu Tea	Green Tea	0.847 ± 0.057	0.276 ± 0.013	37.778 ± 0.481	-	-	-
16	Taiping Houkui Tea	Green Tea	0.761 ± 0.043	0.241 ± 0.013	29.493 ± 0.346	-	0.347 ± 0.007	-
17	Xihu Longjing Tea	Green Tea	0.931 ± 0.043	-	38.508 ± 0.117	2.069 ± 0.097	-	-
18	Yongxi Huoqing Tea	Green Tea	1.060 ± 0.037	0.262 ± 0.011	30.783 ± 0.482	1.799 ± 0.026	-	-
19	Fenghuang Shuixian Tea	Oolong Tea	3.284 ± 0.141	-	34.770 ± 0.138	1.880 ± 0.062	1.185 ± 0.079	-
20	Luohan Chenxiang Tea	Oolong Tea	0.696 ± 0.061	0.232 ± 0.006	30.083 ± 0.287	-	0.572 ± 0.047	-
21	Tieguanyin Tea	Oolong Tea	0.294 ± 0.021	0.176 ± 0.015	14.842 ± 0.167	-	-	-
22	Wuyi Rock Tea	Oolong Tea	2.383 ± 0.142	-	25.881 ± 0.335	-	-	0.545 ± 0.011
23	Gongmei White Tea	White Tea	2.179 ± 0.038	-	27.466 ± 0.059	-	0.498 ± 0.015	-
24	Shoumei White Tea	White Tea	2.022 ± 0.026	-	25.303 ± 0.035	-	0.357 ± 0.021	-
25	White Peony Tea	White Tea	2.486 ± 0.026	-	28.758 ± 0.033	-		-
26	Huoshan Large Yellow Tea	Yellow Tea	3.822 ± 0.111	0.241 ± 0.009	34.201 ± 0.036	3.326 ± 0.037	0.562 ± 0.031	-
27	Junshan Yinzhen Tea	Yellow Tea	0.940 ± 0.019	-	41.457 ± 0.322	1.882 ± 0.052	1.051 ± 0.045	-
28	Mengding Huangya Tea	Yellow Tea	1.495 ± 0.073	0.313 ± 0.007	36.022 ± 0.166	3.357 ± 0.065	0.499 ± 0.030	-
29	Weishan Maojian Tea	Yellow Tea	0.752 ± 0.038	0.249 ± 0.009	37.348 ± 0.220	1.849 ± 0.039	0.688 ± 0.035	-
30	Yuan’an Luyuan Tea	Yellow Tea	0.929 ± 0.031	0.273 ± 0.007	40.737 ± 0.116	2.190 ± 0.023	1.076 ± 0.036	-

DW, dry weight, “-“ means not detected.

**Table 9:** Comparison among natural products regarding FRAP, TEAC, and TPC values.

Index	Natural Product	MIN	Q_L_	M	Q_U_	MAX	Reference
FRAP (μmol Fe(II)/g)	30 Chinese Teas (dry)	611.2	1107.8	2156.9	3465.6	5375.2	This study
	223 Medicinal Plants (dry)	0.1	19.6	65.3	158.4	1844.9	[26]
	34 Fruit Seeds (fresh)	0.3	5.5	11.3	16.1	181.4	[36]
	48 Fruit Peels (fresh)	0.0	6.1	14.2	27.3	155.7	[36]
	56 Wild Fruits (fresh)	1.3	12.9	40.3	135.8	502.0	[37]
	49 Macro-fungi (dry)	7.9	15.1	22.1	34.5	204.7	[38]
	51 Flowers (fresh)	0.2	16.2	27.6	70.0	660.2	[39]
	10 Grape Seeds (fresh)	312.4	357.0	497.3	671.7	858.1	[18]
	30 Grape Peels (fresh)	18.3	59.9	99.9	131.9	253.0	[18]
	30 Grape Pulps (fresh)	1.3	2.9	4.9	6.7	11.8	[40]
	62 Fruits (fresh)	0.1	3.9	6.7	10.2	72.1	[24]
	56 Vegetables (fresh)	2.7	6.9	10.1	13.7	60.9	[23]
TEAC (μmol Trolox/g)	30 Chinese Teas (dry)	326.3	655.9	1396.7	2046.0	3004.4	This study
	223 Medicinal Plants (dry)	1.0	23.5	55.8	116.6	1544.4	[26]
	34 Fruit Seeds (fresh)	2.5	7.6	12.5	19.1	92.6	[36]
	48 Fruit Peels (fresh)	0.0	6.7	14.2	28.6	93.1	[36]
	56 Wild Fruits (fresh)	3.4	17.6	32.7	114.8	1140.0	[37]
	49 Macro-fungi (dry)	4.7	8.6	9.8	20.3	85.7	[38]
	51 Flowers (fresh)	0.2	8.0	12.8	35.1	191.8	[39]
	10 Grape Seeds (fresh)	207.8	227.6	274.5	345.8	473.5	[18]
	30 Grape Peels (fresh)	5.2	27.8	50.4	64.2	123.7	[18]
	30 Grape Pulps (fresh)	0.3	1.1	1.9	2.6	4.8	[40]
	62 Fruits (fresh)	0.8	2.4	3.6	5.0	80.7	[24]
	56 Vegetables (fresh)	6.9	10.3	12.8	15.3	33.6	[23]
TPC (mg GAE/g)	30 Chinese Teas (dry)	37.3	92.7	163.8	202.5	254.3	This study
	223 Medicinal Plants (dry)	0.2	3.8	8.1	14.4	98.9	[26]
	34 Fruit Seeds (fresh)	0.3	2.8	3.7	4.8	23.0	[36]
	48 Fruit Peels (fresh)	0.4	3.5	4.2	6.5	23.0	[36]
	56 Wild Fruits (fresh)	0.5	1.9	6.1	15.8	54.8	[37]
	49 Macro-fungi (dry)	2.4	4.0	4.9	6.5	44.8	[38]
	51 Flowers (fresh)	0.6	3.4	4.9	8.0	36.7	[39]
	10 Grape Seeds (fresh)	34.6	37.3	47.2	59.4	71.2	[18]
	30 Grape Peels (fresh)	1.6	6.3	10.6	13.2	25.7	[18]
	30 Grape Pulps (fresh)	0.3	0.6	0.8	1.0	1.4	[40]
	62 Fruits (fresh)	0.1	0.3	0.6	0.8	5.9	[24]
	56 Vegetables (fresh)	5.0	6.7	7.8	9.4	23.3	[23]

Abbreviations: FRAP, ferric-reducing antioxidant power; M, median; MAX, the maximum value; MIN, the minimum value; Q_L_, the lower quartile; Q_U_, the upper quartile; TEAC, Trolox equivalent antioxidant capacity; TPC, total phenolic content.
